# Impact of multi-limb oscillometric cuff measurements on hemodynamics: insights from pulse wave propagation modeling

**DOI:** 10.3389/fphys.2025.1642645

**Published:** 2025-08-15

**Authors:** Kamil Wolos, Leszek Pstras, Malgorzata Debowska, Jan Poleszczuk

**Affiliations:** Laboratory of Mathematical Modeling of Physiological Processes, Nalecz Institute of Biocybernetics and Biomedical Engineering, Polish Academy of Sciences, Warsaw, Poland

**Keywords:** cardiovascular modeling, oscillometric measurement, hemodynamics, blood pressure, pulse wave propagation

## Abstract

**Objective:**

Multi-limb oscillometric cuff measurements can be used for estimating various vascular parameters and evaluating side differences in arterial pulse waveforms. In this study, we conduct an *in silico* investigation to evaluate the potential impact of such measurements on hemodynamics.

**Methods:**

We employed a 0–1D model of pulse wave propagation to examine the relationship between different levels of oscillometric cuff pressure applied simultaneously at multiple sites (right above the wrists and/or ankles) and the resulting changes in blood pressure and flow at selected sites in the vascular system, assuming the absence of cardiovascular regulatory mechanisms. The simulations included various combinations of cuff placements, including four cuffs applied simultaneously on all limbs. In addition, we conducted both global and local sensitivity analysis to evaluate the impact of selected cardiovascular parameters on the simulation results.

**Results:**

In the case of cuffs placed on all four limbs and inflated to suprasystolic pressure - effectively occluding the vessels beneath the cuffs - our simulations indicated an increase in mean arterial pressure (MAP) of approximately 10% in the ascending aorta, left common carotid artery, and abdominal aorta. Additionally, the mean carotid artery blood flow increased by approximately 11% compared to baseline value. In contrast, for the case with a cuff placed only on one wrist, we observed a significantly smaller MAP increase of only 2.5%, with a 3% rise in mean carotid artery flow. Our sensitivity analysis revealed that these changes can be mitigated by relatively small adjustments in specific cardiovascular parameters, suggesting that properly functioning physiological regulatory mechanisms should easily compensate for the cuff induced hemodynamic alterations. Furthermore, global sensitivity analysis demonstrated that relatively similar increases in MAP and mean carotid blood flow are expected for different combinations of cardiovascular parameters values, indicating the robustness of our findings.

**Significance:**

This *in silico* study suggests that multi-limb cuff-based measurements may induce measurable central hemodynamic alterations if not counteracted by cardiovascular regulatory mechanisms. This suggests that such measurements may not be innocuous to individuals with some deficiencies in cardiovascular regulation. Further investigation is warranted to verify this *in vivo* and, if necessary, establish appropriate safety protocols.

## 1 Introduction

Oscillometric blood pressure (BP) measurement is a widely used non-invasive technique, leveraging cuff-induced arterial occlusion and cuff pressure oscillations to estimate systolic and diastolic arterial blood pressure (Muntner et al., 2019). The most common version of this method involves relatively rapid inflation of a cuff wrapped around a limb (usually around the upper arm) to suprasystolic pressure, temporarily occluding blood flow in the underlying blood vessels. During subsequent cuff deflation, the gradual arterial reopening generates oscillations in the cuff pressure that correlate with pulsatile arterial blood volume changes (Alpert et al., 2014) and form the basis for blood pressure estimation. While the traditional oscillometric method uses a single-cuff measurement with relatively continuous inflation and deflation of the cuff, devices such as AngE (SoT Medical, Austria) utilize simultaneous multi-limb cuff measurements with a more stepped deflation process and longer periods of maintaining the cuff inflated for advanced cardiovascular diagnostics, especially for estimating various vascular parameters and evaluating side differences in arterial pulse waveforms. However, concurrent occlusion of peripheral arteries in multiple limbs for longer periods of time raises questions about hemodynamic interference, particularly in populations with deficiencies in cardiovascular regulation.

Previous studies suggested that arterial occlusion using a cuff on one or two upper limbs can transiently affect central hemodynamics. Liang et al., in an *in silico* study (Liang et al., 2013), demonstrated that the pulse waveform proximal to a single cuff placed around the brachial artery can vary significantly with cuff pressure. Trachet et al. also demonstrated *in silico* local changes in the brachial artery pulse waveform associated with single cuff-induced occlusion (Trachet et al., 2010). However, none of the aforementioned *in silico* studies looked directly at central blood flow and pressure changes following peripheral cuff-induced occlusions. On the other hand, Kashyap et al. found *in vivo* that bilateral brachial artery occlusion, achieved by inflating cuffs on both arms, can lead to changes in carotid and vertebral artery blood flow in asymptomatic patients with a complete circle of Willis (Kashyap et al., 2005). This underscores the potential impact of peripheral vascular flow alterations on cerebral hemodynamics, which is particularly relevant in clinical contexts where maintaining stable cerebral perfusion is crucial. For example, cerebral blood flow alterations are critically important in patients with severe traumatic brain injury (sTBI), as they can cause secondary brain damage and negatively influence long-term outcomes (Graves et al., 2015). A key concern in sTBI is the frequent impairment of cerebrovascular autoregulation, which has been reported in over 70% of patients (Sviri et al., 2009), which renders cerebral blood flow pressure passive and heightens vulnerability to systemic blood pressure fluctuations (Toth et al., 2016; Rangel-Castilla et al., 2008). Moreover, evidence suggests that, in addition to changes in baroreflex sensitivity (BRS) occurring directly after brain trauma (Uryga et al., 2023), both mild and severe TBI can lead to persistent autonomic dysfunction, manifesting as altered heart rate variability, reduced BRS, and impaired cardiovascular responses to physiological stressors such as posture changes or Valsalva maneuver (Hilz et al., 2011; Hilz et al., 2017; Hilz et al., 2016). These impairments have been linked to increased morbidity and mortality in acute brain injury patients (Hilz et al., 2015; Papaioannou et al., 2008; Sykora et al., 2016). Additionally, studies have shown that heart rate responses to both lowering and elevating blood pressure are depressed by propofol anesthesia (Sato et al., 2005), which is frequently administered in intensive care units during the days following severe brain injury to help manage intracranial pressure.

The purpose of our study was to analyze the potential hemodynamic consequences of multi-limb oscillometric measurements using a physiology-based modeling approach. Primarily, we sought to estimate the effect of multi-site cuff inflation on hemodynamic parameters such as mean arterial pressure (MAP) and blood flow at various locations in the arterial tree (with a particular focus on the central arteries), assuming the absence of cardiovascular regulatory mechanisms. For this purpose, we applied a previously developed cardiovascular model of pulse wave propagation (Poleszczuk et al., 2018a; Poleszczuk et al., 2018b; Wołos et al., 2024), which we here adapted to simulate the effects of oscillometric cuffs placed right above the wrists and/or ankles.

To our knowledge, no previous *in silico* study has examined the effects of multiple cuff-induced occlusions on central hemodynamics. We hypothesized that occlusion of arteries at wrists and ankles, in the absence of regulatory mechanisms, may significantly influence blood flow and blood pressure at other sites.

## 2 Materials and methods

### 2.1 Cardiovascular model

In this study, we utilized a previously developed and validated 0-1D cardiovascular model; for more details, see (Poleszczuk et al., 2018a; Poleszczuk et al., 2018b; Wołos et al., 2024) and Supplementary Material S1. The modeled arterial network consists of 71 axisymmetric elastic vessel segments with tapered walls. Blood was modeled as an incompressible Newtonian fluid with a constant density and viscosity. At arterial bifurcations, pressure continuity and flow conservation were imposed. Blood flow was governed by equations derived from the Navier-Stokes equations, describing changes in blood flow, arterial cross-sectional area, and transmural pressure over time. For the inflow boundary condition, we used a time-varying elastance model of the left heart ventricle, whereas the outflow from the terminal arteries of our arterial tree was modeled using 3-element Windkessel models. In Supplementary Material S1, we present the values of all model parameters used in our baseline simulations.

### 2.2 Cuff modeling

In our 1-D model of the arterial tree, we use an elastic model of the arterial wall to describe the relationship between arterial cross-sectional area and transmural pressure (see Supplementary Material S1). However, when a cuff is inflated to a pressure above the arterial blood pressure, the transmural pressure in the arteries under the cuff becomes negative, leading to the so-called vessel collapse (cross-sectional area approaching zero), which cannot be described by the elastic model. To address this, we used indirectly the model proposed by Drzewiecki et al. (1994), which combines the model of elastic distention of arterial wall with the model of its collapse at negative transmural pressures, thus describing the (static) arterial cross-sectional area ( A ) for a wide range of transmural pressures ( $P_{T}$ ) as follows:

$$A=d\frac{\ln\left(aP_{T}+b\right)}{1+\exp\left(-cP_{T}\right)}$$ (1)

where a,b,c , and d are empirical constants.

For simplicity, we used the above relationship only for the arterial segments under the considered cuffs. Moreover, to maintain the computational tractability of our 1-D model of the arterial network, we did not use the above (somewhat complex) relationship explicitly in those arterial segments, but we kept there our standard elastic model, which for each simulation (for a given cuff pressure) was scaled so that it would approximate locally the above relationship around the new expected mean arterial transmural pressure (assuming that the transmural pressure is reduced by an amount equal to the cuff pressure) - see Supplementary Material S1 for further details. This approach allowed us to keep the original structure of our model, while effectively describing the arteries under the cuffs using the appropriate (approximated) parts of the relationship proposed by Drzewiecki et al.

Note that the Equation 1 describes steady-state conditions of an artery, thus disregarding its viscoelasticity. However, in our study we did not attempt to model in detail the process of cuff inflation/deflation (for which viscoelasticity would be particularly important). Instead, we were interested only in steady-state conditions following inflation of cuff(s) to a given pressure and maintaining this pressure for some time (as, for example, in the aforementioned AngE device). For each considered cuff pressure (treated as a parameter in our model), we ran the simulation until a steady state was reached and we reported the results for that steady state to provide an estimate of what could happen in terms of hemodynamics in the worst-case scenario (with no cardiovascular regulation).

In our simulations, oscillometric cuffs were modeled at four locations: the wrists and ankles. At the wrist, we considered a cuff placed above the distal parts of the radial, interosseous, and ulnar arteries. At the ankles, the cuff pressure was applied to the distal parts of the anterior and posterior tibial arteries. We assumed that all cuffs were 15 cm wide (the size of the medium cuffs of the AngE device). Given the unequal lengths of terminal arteries in the considered cuff locations (e.g., the anterior and posterior tibial arteries), we assumed that each cuff was located so that it ended at the distal end of the longest artery. For example, with a 15 cm wide cuff and a 2.5 cm difference in length between the anterior and posterior tibial arteries, the posterior tibial artery (shorter) was covered at its distal end by 12.5 cm of cuff. To ensure that cuff pressure was exerted only on the arterial segments directly under the cuff (i.e., not along the entire length of the underlying arteries), each artery under the considered cuff locations was divided into two segments: one located under the cuff and the other before the cuff. In the arterial segments under the cuffs, the arterial blood pressure was the sum of transmural pressure and the cuff pressure (we assumed that the cuff pressure is transmitted entirely to the underlying arterial segments), whereas in the preceding arterial segments and in all other arteries in our model, the arterial blood pressure was equal to the transmural pressure (for simplicity, we assumed the same zero external pressure for all arteries). All simulations assumed a uniform pressure distribution across the entire cuff width, with the same cuff pressure applied simultaneously to all arterial segments covered by the cuff(s).

### 2.3 Workflow

**FIGURE 1:**
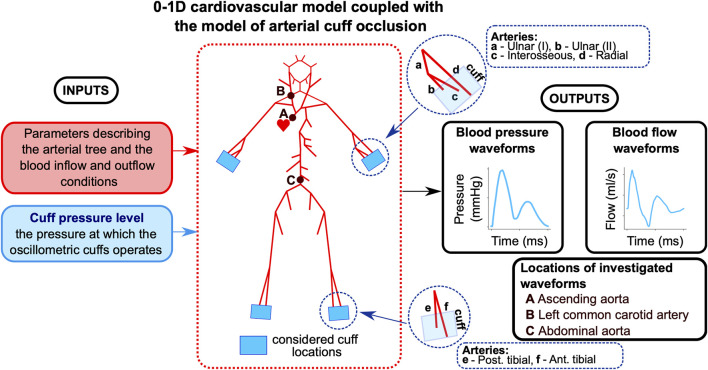
Workflow of the study. The model describes pulse wave propagation in the arterial tree with cuff-induced peripheral arterial occlusion (above wrists and/or ankles), assuming no cardiovascular regulatory mechanisms. The baseline model uses parameter values describing the cardiovascular tree of a 25-year-old healthy man. Arterial pressure and blood flow waveforms are analyzed at the indicated locations.

For baseline simulations, we assumed parameter values representing a 25-year-old healthy male with a height of 175 cm (see Supplementary Material S1). First, we simulated a single cuff scenario (with the cuff above the left wrist) and analyzed the local hemodynamic effects of cuff inflation by assessing the waveforms representing pressure and cross-sectional area in three locations in the radial artery beneath or before the cuff (for different cuff pressure levels). Then, we simulated various multi-cuff scenarios (again, for different cuff pressure levels) and obtained model-predicted waveforms of blood pressure and flow at three central locations within the arterial tree (in the ascending aorta, left common carotid artery, and abdominal artery; see Figure 1). We then compared the simulation results obtained for different cuff pressure levels to assess the impact of varying arterial occlusion levels for different cuff configurations. In all simulations, the same cuff pressure ( $P_{\text{cuff}}$ ) was applied simultaneously to all cuffs considered in the given scenario. We considered cuff pressures from 0 mmHg to 150 mmHg (in 10 mmHg steps). We did not analyze cases with cuff pressures above 150 mmHg, given that the results of our simulations reached a plateau for a cuff pressure of 140 mmHg, and higher cuff pressures caused numerical instability of the calculations due to the cross-sectional area of compressed arteries approaching zero. The cuff pressure of 150 mmHg was 25 mmHg higher than the systolic pressure of our baseline virtual patient, thus leading to near complete occlusion of the arteries.

## 3 Results

### 3.1 Local effects of cuff occlusion

**FIGURE 2:**
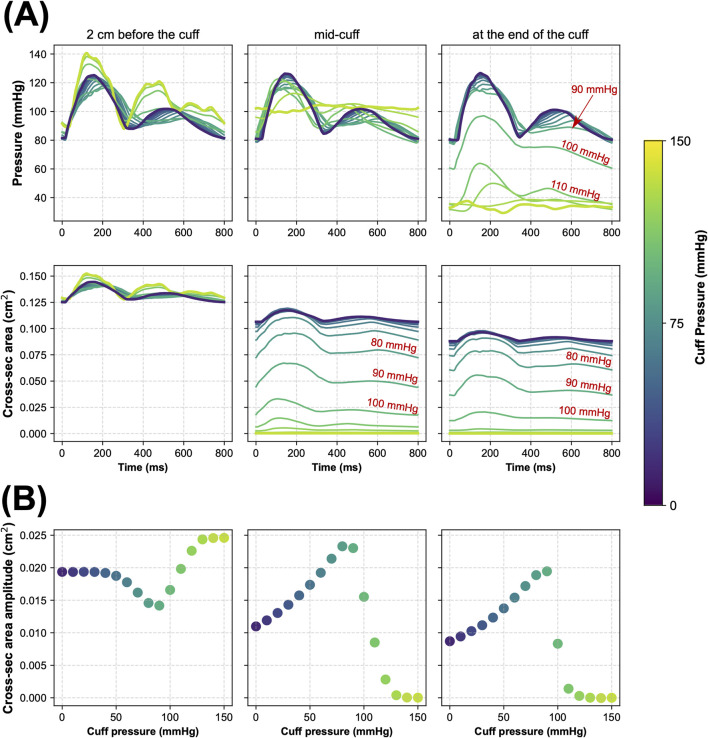
Simulated local hemodynamic effects of cuff-induced arterial occlusion above the left wrist (in the radial artery). **(A)** Blood pressure and cross-sectional area of the radial artery during one cardiac cycle simulated for different levels of cuff pressure in three locations of the radial artery before and under the cuff, i.e., 2 cm before the cuff (left panels), in the middle of the cuff (middle panels), and at the end of the cuff (right panels). **(B)** The amplitude of cross-sectional area pulsations of the radial artery simulated for different cuff pressure levels in the same three locations of the radial artery.

Figure 2 illustrates how inflation of the cuff placed above the wrist affects pressure and cross-sectional area waveforms at three locations in the radial artery relative to the cuff: 1) proximal (2 cm before the cuff), 2) in the middle of the cuff, and 3) distal (at the end of the cuff). Similar waveform alterations were observed for other arteries beneath the cuff (i.e., the interosseous and ulnar arteries).

When $P_{\text{cuff}}$ remained below diastolic pressure (DP; around 80 mmHg), the pressure waveforms exhibited relatively small changes. Significant pulse pressure waveform alterations began to appear around $P_{\text{cuff}}$ = 90 mmHg, particularly for the distal segment of the radial artery. The arterial cross-sectional area under the cuff decreased with rising cuff pressure, while the amplitude of its pulsations peaked for cuff pressure near MAP level (95 mmHg), which is an expected effect that is used in the classic oscillometric BP measurement (Drzewiecki et al., 1994). Noticeable changes in the arterial cross-sectional area proximal to the cuff began to appear once $P_{\text{cuff}}$ exceeded the MAP level, mirroring pressure waveform alterations. Virtually complete vessel occlusion (>99% reduction in mean blood flow) occurred for $P_{\text{cuff}}$ of 130 mmHg, i.e., when $P_{\text{cuff}}$ exceeded the systolic pressure (SP; 125 mmHg), as evidenced by the virtually zeroed cross-sectional area waveforms beneath the cuff.

### 3.2 Central effects of multi-limb cuff occlusion

**FIGURE 3:**
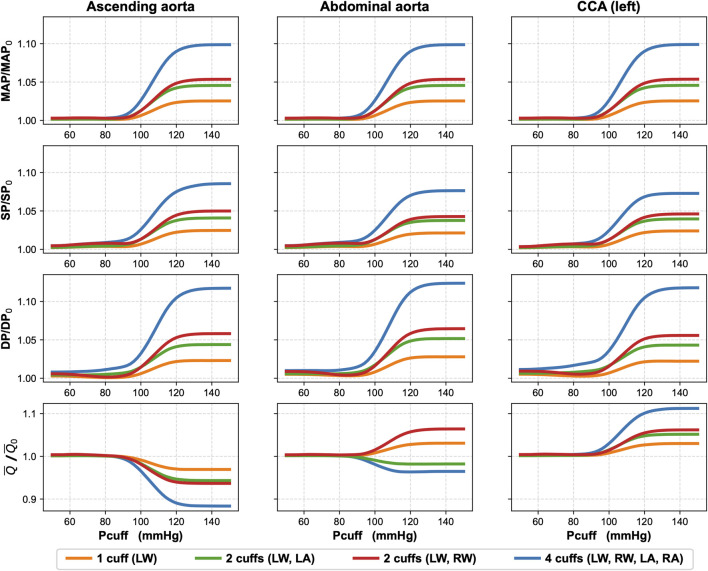
Effects of single and multi-limb cuff occlusions on blood pressure and blood flow in three arteries: ascending aorta, abdominal aorta, and left common carotid artery (CCA). The simulations present relative changes in mean arterial pressure (MAP), systolic pressure (SP), diastolic pressure (DP), and mean blood flow ( $\bar{Q}$ ) in the three considered arteries for different cuff pressure levels ( $P_{cuff}$ ), compared to baseline values (i.e., for $P_{cuff}$ = 0). Various combinations of cuff occlusions were considered. LW and RW denote the left and right wrist, respectively, and LA, RA denote the left and right ankle, respectively. Subscripts 0 indicate baseline values.

For simultaneous four-limb total arterial occlusion ( $P_{\text{cuff}}$ = 150 mmHg), our simulations demonstrated a 9.9% increase in MAP in the ascending aorta, left common carotid artery (CCA), and abdominal aorta relative to baseline values ( $P_{\text{cuff}}$ = 0 mmHg); see Figure 3. At the same time, mean carotid artery blood flow increased by 11.2% compared to baseline, while the mean blood flow in the abdominal aorta and ascending aorta decreased by 3.5% and 11.6%, respectively, relative to baseline. Similar changes were observed for all cuff pressure levels above the baseline systolic pressure (i.e., above 125 mmHg).

We also performed additional simulations to assess the impact of total arterial occlusion with other cuff placement configurations. In Figure 3 we show the results for unilateral (left) wrist occlusion, bilateral wrist occlusion, and hemilateral occlusion (left wrist and left ankle). Due to the relatively symmetrical structure of our arterial model (complete symmetry in the case of the arteries in the legs and close to symmetry in the case of the arms), right-sided occlusions yielded analogous results. Across all analyzed cuff configurations, a consistent trend was observed: when cuff pressure remained below the baseline MAP, central hemodynamic changes were negligible. Significant changes in blood pressure and mean blood flow in the analyzed central arteries began to appear when cuff pressure approached and exceeded the baseline MAP (95 mmHg).

Upper-limb total occlusion (bilateral wrist cuffs) induced a 6.4% increase in the abdominal aorta blood flow toward the lower limbs relative to the baseline value. Conversely, left-sided hemilateral total peripheral occlusion reduced abdominal aorta flow by 1.8% compared to baseline. The mean blood flow in the left CCA increased by 6.2% and 5.2% for the total occlusion by upper-limb cuffs and left-limb cuffs, respectively. These changes were less pronounced than those observed during four-limb total occlusion. Accordingly smaller changes were observed for unilateral (left) wrist total occlusion, which resulted in approximately fourfold smaller changes than during the four-limb total occlusion. Specifically, in the case of single wrist occlusion, MAP in the ascending aorta, abdominal aorta, and left CCA increased by only 2.5% relative to baseline, while the mean blood flow in the left CCA rose by only 3%.

### 3.3 Sensitivity analysis

**FIGURE 4:**
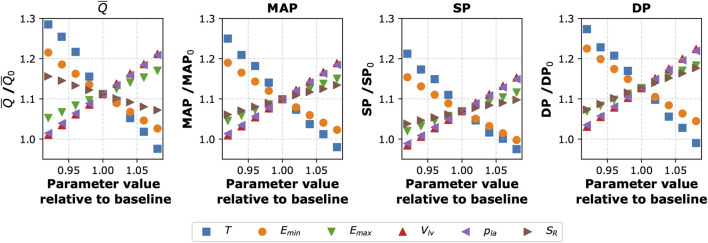
Sensitivity of selected model outputs to the combined effect of inflating the cuffs to 150 mmHg (on all four limbs) and changing the values of selected (single) model parameters. The y-axis shows the percentage of the given model output relative to baseline value (without the cuffs and with the baseline parameter values). The considered model outputs (for the left common carotid artery): $\bar{Q}$ – mean blood flow, MAP–mean arterial pressure, SP–systolic pressure, DP–diastolic pressure; The tested model parameters: T–heart period, $E_{\min},E_{\max}$ – minimal and maximal left ventricular elastance, $V_{lv}$ – left ventricular end-diastolic volume, $p_{la}$ – left atrial pressure, $S_{R}$ – scaling factor for resistances of small arteries and arterioles (terminal segments of the arterial tree). Subscript 0 indicates baseline values (i.e., simulations for $P_{cuff}$ = 0 and baseline parameter values).

To assess how changes in the values of selected cardiovascular parameters could counteract the central hemodynamic changes caused by the cuff-induced arterial occlusions, we conducted a local sensitivity analysis. Namely, we studied the sensitivity of selected model outputs to the combined effect of inflating the cuffs to 150 mmHg and changing locally the values of selected (single) model parameters. The analyzed model outputs included blood pressure (SP, DP, and MAP) and mean blood flow ( $\bar{Q}$ ) in the left CCA. We have focused on the artery supplying blood to the brain, as blood pressure and blood flow in this artery may be crucial in patients with impaired cerebral autoregulation. Each of the studied parameters (see Supplementary Material S1 for a list of the parameters considered in the sensitivity analysis) was varied within ±10% of its default value (with other parameters unchanged), and the corresponding changes in the analyzed model outputs were expressed in relation to their baseline values. Our analysis revealed that the two considered model outputs are most sensitive to variations in the following cardiovascular parameters: heart period ( T ), minimal and maximal left ventricular elastance ( $E_{\min}$ and $E_{\max}$ ), end-diastolic left ventricular volume ( $V_{lv}$ ), left atrial pressure ( $p_{la}$ ), and the scaling factor for terminal arterial resistances ( $S_{R}$ ); see Figure 4.

**FIGURE 5:**
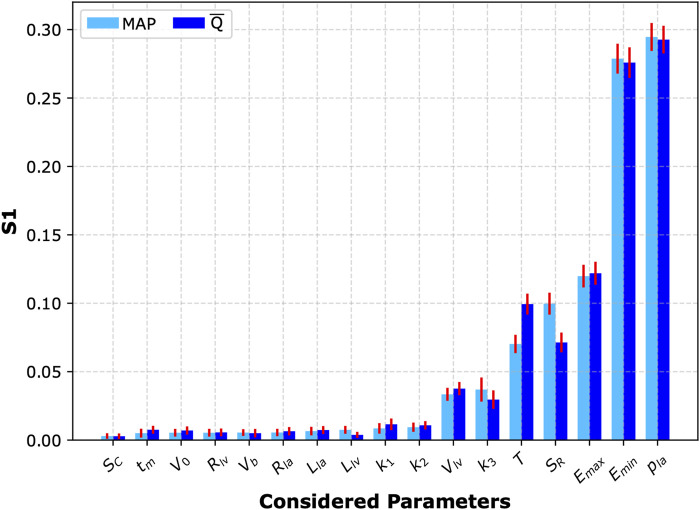
Sobol’ sensitivity analysis. First-order Sobol’ indices (S1) with 95% confidence intervals (red bars) representing sensitivity of the two analyzed model outputs (mean arterial pressure, MAP, and mean blood flow in the left common carotid artery, $\bar{Q}$ ) to changes in the values of selected cardiovascular parameters (during four-limb cuff occlusion). The considered parameters included: $S_{C}$ – scaling factor for compliances of small arteries and arterioles, $t_{m}$ – time to the onset of the constant left ventricular elastance, $V_{0}$ – volume of the left ventricle at zero transmural pressure, $R_{lv}$ – resistance against blood flow from the left ventricle to the ascending aorta, $V_{b}$ – volume of the backflow from the ascending aorta to the left ventricle, $R_{la},L_{la}$ – resistance and inertia terms for the blood flow from the left atrium to the left ventricle, respectively, $L_{lv}$ – inertia term for the blood flow between the left ventricle and ascending aorta, $k_{1},k_{2},k_{3}$ - parameters describing stiffness of the small, medium and large arteries, respectively, $V_{lv}$ – left ventricular end-diastolic volume, T – heart period, $S_{R}$ – scaling factor for resistances of small arteries and arterioles (terminal segments of the arterial tree), $E_{\max},E_{\min}$ – maximal and minimal left ventricular elastance, $p_{la}$ – left atrial pressure.

To investigate the impact of simultaneous variations in all analyzed cardiovascular parameters on mean blood flow and MAP in the left CCA following the four-limb cuff occlusion, we also performed a global sensitivity analysis for all considered parameters. To this end, we computed first-order Sobol’ indices (Sobol′, 2001; Saltelli et al., 2010) based on 8,000 simulations of the four-limb cuff occlusion (again with $P_{\text{cuff}}$ = 150 mmHg) with different sets of cardiovascular parameter values sampled randomly from a uniform distribution within ±10% of their default values. This approach allowed us to quantify the contribution of each parameter to the total variance in the analyzed model outputs (i.e., either MAP or mean blood flow in the left CCA following the four-limb total cuff occlusion). This analysis showed that the parameters with the most significant impact on the studied model outputs are almost identical to those identified in the local sensitivity analysis, see Figure 5.

**FIGURE 6:**
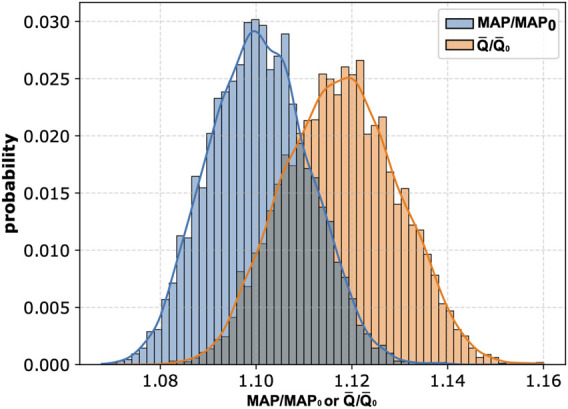
Histograms of relative changes in mean arterial pressure (MAP) and mean blood flow in the left common carotid artery ( $\bar{Q}$ ) following peripheral arterial occlusion simulated for 8,000 combinations of cardiovascular parameter values. Parameter values were sampled within ±10% of their default values. Simulations were performed for the case with four cuffs placed above the wrists and ankles and inflated to 150 mmHg. Subscript 0 indicates baseline values (i.e., simulations without cuff occlusions).

It is also worth noting that the magnitude of potential changes in MAP and mean carotid blood flow induced by peripheral arterial occlusion by cuffs (assuming the absence of cardiovascular regulation) can depend on the baseline values of cardiovascular parameters, which are naturally subject to inter-patient variability. For instance, older individuals typically exhibit increased arterial stiffness (Vatner et al., 2021), compared to younger individuals. To explore this inter-patient variability, we performed another global sensitivity analysis with 8,000 pairs of simulations (i.e., a baseline simulation without cuffs and a simulation with cuffs on all four limbs inflated to 150 mmHg) using different combinations of cardiovascular parameter values randomly sampled from a ±10% range around their default values. The histograms illustrating the distributions of relative changes in MAP and mean blood flow in the left CCA across these 8,000 simulations are presented in Figure 6. On average, these simulations demonstrated an approximate 10% increase in MAP and a 12% increase in mean blood flow in the left CCA relative to baseline values.

## 4 Discussion

In this study, we employed a pulse wave propagation model to investigate the hemodynamic effects of cuff-induced peripheral arterial occlusion at single or multiple limbs, assuming the absence of cardiovascular regulatory mechanisms and considering steady-state conditions after inflating the cuff(s) to a given pressure. While previous mathematical modeling studies have investigated single cuff mechanics (Liang et al., 2013; Trachet et al., 2010; Liang et al., 2012), to our knowledge, this is the first study to analyze the cumulative impact of simultaneous multi-limb cuff occlusion, as used for advanced cardiovascular diagnostics. Our *in silico* study demonstrates that multi-limb cuff-based measurements, if not counteracted by the properly functioning cardiovascular regulatory mechanisms, can alter MAP and the central blood flow distribution. As expected, the most pronounced changes were observed when cuffs were placed on all four limbs and inflated to suprasystolic pressure. Specifically, we observed an increase in MAP of approximately 10% in our baseline virtual patient and up to approximately 14% in the analyzed cohort of virtual patients (see Figure 6). It should be emphasized that this represents a theoretical “worst-case” scenario, as our model intentionally did not account for cardiovascular regulation. It is to be expected that properly functioning regulatory mechanisms should compensate the effects of cuff occlusion, as has been shown in an *in-vivo* study in healthy subjects (Kashyap et al., 2005).

The proposed model seems to reliably reflect the local physiological responses to cuff inflation. As illustrated in Figure 2, the higher the cuff pressure, the higher the reduction in the cross-sectional area of the arteries under the cuff (represented by the radial artery for the wrist cuff scenario). Concurrently, the maximal amplitude of the pulsations of the arterial cross-sectional area was observed for $P_{\text{cuff}}\approx$ MAP. This aligns with the known relationship between arterial compliance and transmural pressure, with the maximal compliance occurring at transmural pressure close to zero (i.e., when $P_{\text{cuff}}\approx$ MAP), which corresponds to the point of mechanical buckling of the arterial wall, where buckling is defined as the transition of the vessel shape form circular to non-circular (Drzewiecki et al., 1994), although in our model we always assume a circular cross-section of the vessels.

Regardless of cuff configuration, for $P_{\text{cuff}}$ values below MAP, relative changes in blood pressure in the central arteries (compared to baseline) were generally negligible, as shown in Figure 3. Bilateral wrist occlusion produced a slightly distinct pattern for different cuff inflation levels, compared to other cuff configurations: first, a minor increase in blood pressure, followed by a slight decrease before the main rise for higher cuff pressures. This could be related to wave reflection and superposition effects, which are modulated by varying degrees of cuff inflation, although, also in this case, the changes in central blood pressure were rather negligible. For cuff pressures above MAP, we observed that the higher the cuff pressure, the lower the mean blood flow in the ascending aorta (up to a plateau for $P_{\text{cuff}}$ = 140 mmHg). This was an entirely expected effect as peripheral arterial occlusions increase systemic vascular resistance, requiring the heart to work against a higher afterload (the phenomenon accounted for in our model), leading to a reduced left ventricular stroke volume and hence reduced cardiac output (assuming the absence of cardiac regulation). The increase in central MAP is caused mainly by the increase in the total systemic vascular resistance but partly also by additional reflection of pressure waves from the cuff occlusion sites (see Figure 2 for these additional reflections visible in arterial pressure waveforms before the cuff when the cuff is inflated to high pressures). In the case with cuff(s) on one or both ankles, the mean blood flow in the abdominal aorta is reduced, despite the increase in central MAP, which can be explained by the increased peripheral resistance in one or both legs, respectively. In contrast, in the case with cuff(s) on one or both wrists, the mean blood flow in the abdominal aorta is increased, because in this case the increase in central MAP is combined with no change in peripheral resistance of the lower body. This increase in the mean blood flow in the abdominal aorta may seem paradoxical, given the reduced cardiac output. This can be explained by redistribution of central blood flow–a higher arterial resistance in the upper limbs translates to relatively higher proportion of cardiac output being directed towards the lower body. For a similar reason, the mean blood flow in the common carotid artery increases in all considered cuff configurations.

As already mentioned, our simulations did not account for cardiovascular regulatory mechanisms (e.g., baroreflex mechanisms or cerebral autoregulation). However, this approach may be physiologically relevant for populations with impaired regulatory mechanisms, such as patients with severe traumatic brain injury (Toth et al., 2016). In such patients (assuming a significant regulatory impairment), the potential increase in cerebral blood flow induced by four-limb cuff inflation to suprasystolic pressure could elevate intracranial pressure, potentially leading to secondary brain injury.

An example of a device utilizing multi-limb oscillometric measurements is AngE (SoT Medical, Austria), which typically employs the following measurement protocol: initial inflation of the cuffs to suprasystolic pressure (180 mmHg by default), followed by cuff pressure reductions by 10 mmHg every 5 s, until 40 mmHg is reached. This means that the cuffs may remain inflated to a suprasystolic pressure for a much longer time compared to the standard blood pressure measurement. Moreover, in clinical practice, such a measurement protocol may be repeated for data averaging, which could therefore lead to repeated increases in blood pressure in patients with impaired cardiovascular regulation, with a possible accumulation of their potentially negative effects. On the other hand, assuming fully functional regulatory mechanisms, changes in the cardiovascular system initiated by the autonomic nervous system (ANS) and the humoral system to counteract the effects of peripheral arterial occlusion induced by cuff inflation may potentially lead to some distortion of the measurement results, i.e., the measured or estimated parameters may not necessarily correspond to resting conditions but may partly reflect the changes that occurred in the cardiovascular system in response to cuff inflation. For instance, devices such as the AngE, provide an average waveform of the recorded cuff pressure fluctuations (reflecting fluctuations in blood volume under the cuff). However, the morphology of these waveforms could be affected by the activation of the ANS following cuff inflation. ANS controls the heart rate, heart contractility, peripheral arterial resistance, and venous compliance, all of which may affect the observed waveform morphology. Similarly, measurements of pulse wave velocity using the time shift between waveforms recorded in wrist and ankle cuffs may be somewhat distorted by changes in heart rate possibly induced by cuff inflation, although there are conflicting results regarding whether the effect of heart rate on arterial stiffness is a pressure-independent phenomenon (Tan et al., 2018).

Our sensitivity analyses revealed that the cardiac function parameters have the largest impact on the central hemodynamic response to cuff inflation. Specifically, the local sensitivity analysis identified heart period ( T ), maximal and minimal left ventricular elastance ( $E_{\min},E_{\max}$ ), and left atrial pressure ( $p_{la}$ ) as the key factors influencing central blood pressure and blood flow following the four-limb cuff occlusion (as assessed in the left CCA). The global sensitivity analysis confirmed these findings, highlighting $p_{la}$ and $E_{\min}$ as the primary determinants of MAP and mean blood flow in the left CCA. This is because $p_{la}$ and $E_{\min}$ directly determine cardiac output by governing the left ventricular preload and compliance, respectively. Since MAP is fundamentally driven by cardiac output, these cardiac-related parameters have a dominant influence on central hemodynamics. While arterial stiffness parameters (such as $k_{3}$ ) also affect central blood pressure, their impact on the MAP is secondary. This suggests that during oscillometric cuff measurements blood pressure may be most effectively controlled by regulating the cardiac function.

## 5 Limitations and directions for future research

This *in silico* study has several limitations. First, we assumed the absence of any cardiovascular regulatory mechanisms (e.g., baroreflex). On the one hand, this allowed us to investigate the potential hemodynamic effects of simultaneous multi-limb arterial cuff occlusion in hypothetical patients with complete cardiovascular regulatory dysfunction. On the other hand, however, we could not investigate the effects of partial impairment (either underactivity or overactivity) of all or selected regulatory mechanisms. Moreover, we used a pulse wave propagation model that involves only the pulsatile outflow of blood from the left heart ventricle and its subsequent flow through the arterial tree, without taking into account microcirculation, venous return, and cardiopulmonary circulation, thus neglecting the closed-loop nature of the cardiovascular system (in our model, the left atrial pressure is assumed constant). This means that the results of our simulations (in particular, the observed increase in central blood pressure and carotid blood flow following peripheral arterial occlusion) should be treated only as potential short-term effects since even in the assumed absence of cardiovascular regulatory mechanisms, an increase in arterial blood volume (associated with an increase in arterial blood pressure), combined with a decrease in cardiac output (manifested by decreased blood flow in the ascending aorta), should lead to a decrease in venous blood volume and therefore a decrease in central venous pressure, ultimately leading to a further decrease in cardiac output, which would counteract the increase in arterial blood pressure (in addition to the decrease in cardiac output due to the increased afterload, which we accounted for). On the other hand, the decrease in the left ventricular stroke volume caused by increased afterload should lead to a transient increase in the left atrial pressure (kept constant in our model), thus limiting to some extent a further decrease in cardiac output, at least transiently. Therefore, even considering the scenario without cardiovascular regulatory mechanisms, the prediction of the central hemodynamic effects of peripheral arterial occlusion is not straightforward without using a full closed-loop model of the cardiovascular system. Furthermore, we only analyzed steady-state conditions following cuff inflation to different cuff pressure levels, thus ignoring arterial viscoelasticity and hence disregarding the time it takes for the arteries under the cuffs to collapse under high (suprasystolic) cuff pressures. We did not model the dynamic cuff inflation/deflation processes or the impact of the duration of maintaining the cuffs inflated to a high pressure. Similarly, we did not analyze the potential cumulative effects of repeated cuff-based measurements as is often the case in clinical protocols. Finally, another limitation of our study is the lack of validation of our cuff occlusion model. Therefore, *in vivo* validation would be, essential to confirm the clinical relevance of our findings. In case these model-based observations are confirmed, future studies should establish appropriate safety protocols for multi-limb cuff-based measurements in vulnerable populations (e.g., in severe traumatic brain injury patients), especially with regard to maximal time of maintaining suprasystolic cuff pressure. However, designing such a validation protocol requires careful ethical consideration. One approach could involve performing the multi-limb cuff measurements in sTBI patients with continuous monitoring of central hemodynamic parameters and intracranial pressure (ICP), but instead of a rapid inflation of the cuffs to a pressure substantially above the systolic pressure, it would be recommended to first inflate the cuffs to a lower pressure (e.g., to a pressure equal to MAP) and then gradually increase the cuff pressure while closely monitoring ICP, so that the test could be quickly aborted in the event of a dangerous rise in ICP. An alternative protocol could involve assessing changes in central hemodynamic parameters following multi-limb cuff inflation in healthy volunteers under pharmacological autonomic blockade to temporarily and reversibly impair cardiovascular regulation.

## 6 Conclusion

We used a pulse wave propagation model to assess the short-term influence of multi-limb cuff occlusion on central hemodynamics in the case of inactive cardiovascular regulatory mechanisms. In such a scenario, according to our simulations, four-limb arterial occlusion can potentially increase MAP by about 10% in the ascending aorta, left common carotid artery, and abdominal aorta, with concurrent central blood flow redistribution (an 11.2% increase in the mean carotid blood flow despite an 11.6% decrease in the ascending aorta blood flow). Our results suggest that simultaneous multi-limb arterial occlusion can lead to noticeable changes in central hemodynamics if not counteracted by properly functioning cardiovascular regulatory mechanisms. This suggests that caution should be exercised when performing this type of measurement in patients with significant impairments in regulatory mechanisms, although further research is required to confirm the clinical relevance of our findings.

## Data Availability

The raw data supporting the conclusions of this article will be made available by the authors, without undue reservation.
