# Supplementary material for: Impact of multi-limb oscillometric cuff measurements on hemodynamics: insights from pulse wave propagation modeling
Source: Front Physiol. 2025 Aug 15;16:1642645. doi: 10.3389/fphys.2025.1642645 (PMC12394220; doi:10.3389/fphys.2025.1642645)
Supplement: Supplementary file 1 [file DataSheet1.pdf]

Supplementary Material 1

1 Cardiovascular model

Here, we provide a brief description of the mathematical model of the cardiovascular system used in our study. A more detailed explanation is available in our previous publications (1,2).

1.1 Arterial tree

The vascular tree considered in this study consists of 71 major human arteries (see Supp. Figure 1). The geometric properties of the considered arteries are listed in Table 1. Each arterial segment is defined by its length L, inlet internal radius r_{in}, and outlet internal radius r_{out}. Arterial segments are modeled as compliant, axisymmetric, tapering cylinders with impermeable walls. The vessel tapering is described by the following equation:

$$r_0(x) = r_{\text{in}} \left(\frac{r_{\text{out}}}{r_{\text{in}}}\right)^{x/L}, \qquad \textbf{(1)}$$

where $r_0(x)$ denotes the internal radius of a given artery at point x at the nominal pressure P_0 (3).

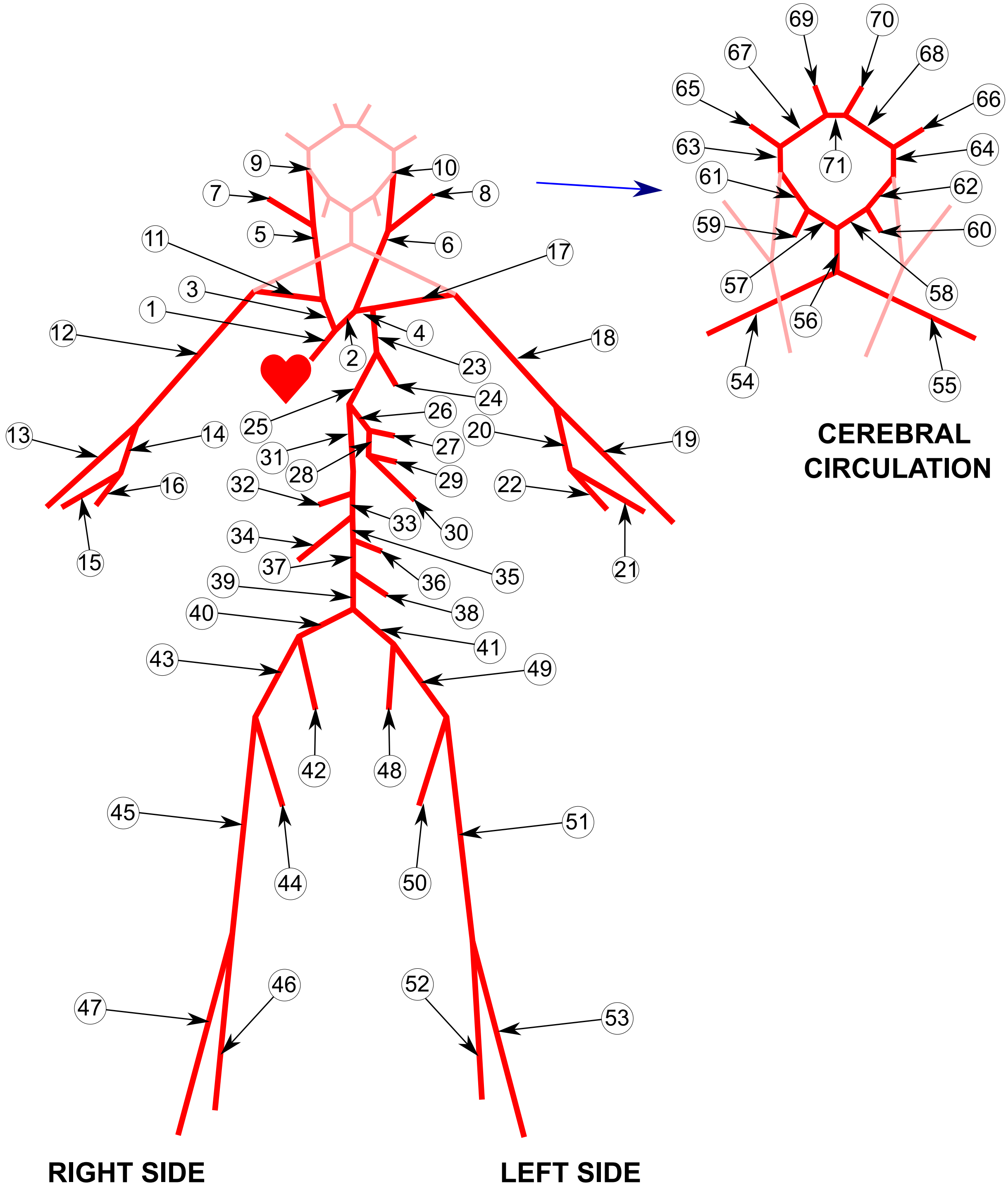

Supp. Figure 1 Overview of the modelled arterial tree. For more information on individual arteries, see **Table 1**.

Table 1 Geometry of the modelled arteries and the corresponding peripheral resistances and compliances. Arterial length (as well as inlet and outlet internal radii ($\boldsymbol{r}_{\text{in}}$ and $\boldsymbol{r}_{\text{out}}$) are provided in centimeters. Peripheral resistance ($\boldsymbol{R_T}$, $\mathbf{10^4}$ g/cm^4/s) and compliance ($\boldsymbol{C_T}$, $\mathbf{10^6}$ cm^{4}s^2/g) are provided only for terminal arteries. L and R stand for left and right, respectively. Data adapted from (4,5).

ID	Artery name	Length	r_{in}	r_{out}	R_T	C_T
1	Ascending aorta	4	1.2	1.18	-	-
2	Aortic arch (I)	2	1.12	1.11	-	-
3	Brachiocephalic	3.4	0.62	0.61	-	-
4	Aortic arch (II)	3.9	1.07	1.06	-	-
5/6	Common carotid R/L	17.7/20.8	0.25	0.25	-	-
7/8	External carotid R/L	17.7	0.15	0.14	5.43	12.7
9/10	Internal carotid (I) R/L	17.7	0.2	0.2	-	-
11/17	Subclavian (I) R/L	3.4	0.42	0.42	-	-
12/18	Subclavian (II) R/L	42.2	0.4	0.24	-	-
13/19	Radial R/L	23.5	0.17	0.14	5.28	3.52
14/20	Ulnar (I) R/L	6.7	0.22	0.22	-	-
15/21	Interosseous R/L	7.9	0.1	0.1	8.40	0.22
16/22	Ulnar (II) R/L	17.1	0.2	0.18	5.28	3.52
23	Thoracic aorta (I)	5.2	1	1	-	-
24	Intercostals	8	0.2	0.15	1.39	13.38
25	Thoracic aorta (II)	10.4	0.68	0.65	-	-
26	Celiac (I)	1	0.39	0.39	-	-
27	Hepatic	6.6	0.22	0.22	3.64	5.13
28	Celiac (II)	1	0.2	0.2	-	-
29	Gastric	7.1	0.18	0.17	5.43	3.44
30	Splenic	6.3	0.18	0.17	2.32	8.01
31	Abdominal aorta (I)	5.3	0.61	0.6	-	-
32	Superior mesenteric	5.9	0.44	0.42	0.93	20.0
33	Abdominal aorta (II)	1	0.6	0.59	-	-
34/36	Renal R/L	3	0.26	0.25	1.13	16.46
35	Abdominal aorta (III)	1	0.59	0.58	-	-
37	Abdominal aorta (IV)	10.6	0.58	0.55	-	-
38	Inferior mesenteric	5	0.17	0.16	6.89	2.7
39	Abdominal aorta (V)	1	0.54	0.52	-	-
40/41	Common iliac R/L	5.8	0.37	0.35	-	-
42/48	Internal iliac R/L	5	0.2	0.19	7.96	2.34
43/49	External Iliac R/L	14.5	0.32	0.27	-	-
44/50	Deep femoral R/L	12.6	0.26	0.19	4.79	3.90
45/51	Femoral R/L	44.5	0.26	0.19	-	-
46/52	Posterior tibial R/L	32.1	0.16	0.14	4.79	3.90
47/53	Anterior tibial R/L	34.3	0.13	0.12	5.60	3.33
54/55	Vertebral R/L	14.8	0.14	0.14	-	-
56	Basilar	3	0.16	0.11	-	-
57/58	Posterior cerebral (I) R/L	0.5	0.11	0.11	-	-

59/60	Posterior cerebral (II) R/L	8.5	0.11	0.11	11.08	6.20
61/62	Posterior communicating R/L	1.5	0.07	0.07	-	-
63/64	Internal carotid (II) R/L	0.5	0.2	0.19	-	-
65/66	Middle cerebral R/L	12	0.14	0.12	5.97	11.60
67/68	Anterior cerebral (I) R/L	1.2	0.12	0.12	-	-
69/70	Anterior cerebral (II) R/L	10	0.12	0.10	8.48	8.20
71	Anterior communicating	0.3	0.07	0.07	-	-

1.2 Blood flow

The equations governing blood flow in the arteries are derived from the incompressible Navier-Stokes equations, assuming constant blood density ρ and viscosity μ, and a Poiseuille velocity profile. These equations describe the flow rate $Q(x,t)$, internal cross-sectional area $A(x,t)$, and transmural pressure $P(x,t)$. A system of equations is formed with the following three equations. The continuity and momentum equations are derived using standard methods (3,6):

$$\frac{\partial Q(t,x)}{\partial x}+\frac{\partial A(t,x)}{\partial t}=0 \quad \textbf{(2)}$$

$$\frac{Q(t,x)}{\partial t}+\frac{\partial}{\partial x}\left(\frac{Q(t,x)}{A(t,x)}\right)+\frac{A(t,x)}{\rho}\frac{\partial P(t,x)}{\partial x}=-\frac{8\pi\mu}{\rho}\frac{Q(t,x)}{A(t,x)} \quad \textbf{(3)}$$

The third equation describes the relationship between arterial cross-sectional area and transmural pressure P_T, assuming the arterial walls are purely elastic (3):

$$P_T(t,x)-P_0=f(x)\left(1-\sqrt{\frac{A_0(x)}{A(t,x)}}\right), \quad \textbf{(4)}$$

where $A_0(x)$ is the arterial internal cross-sectional area at point x at nominal pressure P_0, i.e., $A_0(x)=\pi r_0^2(x)$, and the function $f(x)$ describes the elasticity of the artery wall as follows:

$$f(x)=\frac{4}{3}\left(k_1\, exp\left(k_2 r_0(x)\right)+k_3\right), \quad \textbf{(5)}$$

where parameter k_1 describes the stiffness of smaller arteries, k_2 reflects the transition between the large, elastic arteries and smaller, less-elastic arteries, and k_3 may be interpreted as the stiffness of large arteries (the vast majority of arteries in our model) (7).

1.3 Model of the cuff inflation

To model the impact of cuff inflation on arteries beneath the cuff, we used a nonlinear relationship between arterial internal cross-section (A_d) and transmural pressure (P_T) proposed by Drzewiecki et al. (8) (following its calibration, as described below):

$$A_d = d\frac{\ln(aP_T + b)}{1 + \exp(-cP_T)}, \quad \textbf{(6)}$$

where $a, b, c,$ and d are empirical constants. This relationship combines the model of elastic distention of arterial wall with the model of its collapse at negative transmural pressures (cross-sectional area approaching zero), thus describing the (static) arterial cross-sectional area for a wide range of transmural pressures.

For simplicity, we used the above relationship only for the arterial segments under the considered cuffs. Moreover, to maintain the computational tractability of our 1-D model of the arterial network, we did not use the above relationship explicitly in those arterial segments, but we kept there our standard elastic model (equation (4)), which for each simulation (for a given cuff pressure) was scaled so that it would approximate locally the above relationship around the new expected mean arterial transmural pressure (assuming that the transmural pressure is reduced by an amount equal to the cuff pressure).

Our methodology involved the following calibration process (see. Supp. Figure 2). First, we calibrated the parameters $a, b, c,$ and d of the Drzewiecki model to closely match our standard elastance model of the arterial wall (as given by Olufsen (3)) within the physiological pressure range, i.e. the range 80-120 mmHg. This ensured alignment with our baseline model under normal conditions. Next, to simulate cuff inflation, we used the Drzewiecki model to predict the reduced arterial cross-sectional area at external pressure corresponding to cuff pressure. Then, for each considered cuff pressure level, we scaled the Olufsen-based elastance model, i.e., we found the values of parameters P_{new} and g modifying equation (4), as follows:

$$P_T(t,x) = P_0 + P_{new} + g \cdot f(x)\left(1 - \sqrt{\frac{A_0(x)}{A(t,x)}}\right), \quad \textbf{(7)}$$

to approximate locally the Drzewiecki model within ± 20 mmHg around the estimated mean transmural pressure (for the given cuff pressure level). This scaling method allowed us to capture the nonlinear behavior of selected arterial segments during cuff occlusion while preserving the computational efficiency and structure of our existing 0-1D model.

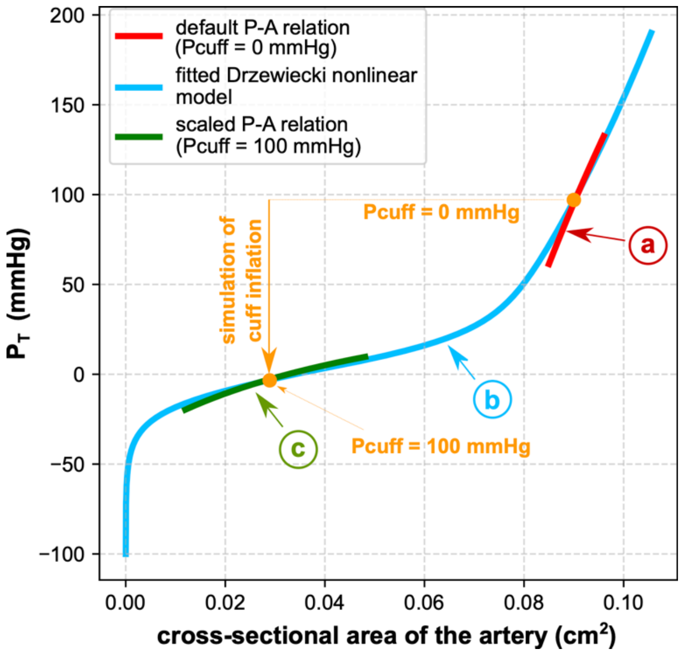

Supp. Figure 2 Calibration of the arterial pressure-area (P-A) relationship to simulate cuff inflation (example provided for the cuff pressure $\boldsymbol{P}_{\mathbf{cuff}}$ = 100 mmHg). The calibration process for a given artery consists of three steps: (a) calibrating the parameters of the Drzewiecki model (equation 6) to fit as closely as possible the default P-A relationship (equation 4, red line) in the physiological pressure range (i.e. the range 80-120 mmHg); (b) using the fitted Drzewiecki model (blue line) to predict the arterial cross-sectional area at the given cuff pressure (assuming that the transmural pressure is reduced by an amount corresponding to the cuff pressure), and (c) scaling the default P-A relationship (equation 4) to approximate locally the Drzewiecki model (green line). By $\boldsymbol{P_T}$ we denoted transmural pressure.

1.4 Arterial bifurcations

At all modeled arterial bifurcations, we assume pressure continuity and mass conservation (i.e., no blood leakage). Let p represent the parent vessel and d_1, d_2 the daughter vessels. The assumed conditions can be expressed as follows:

$$P_{\text{out},p} = P_{\text{in},d_1} = P_{\text{in},d_2} \quad \text{and} \quad Q_{\text{out},p} = Q_{\text{in},d_1} + Q_{\text{in},d_2}, \qquad \textbf{(8)}$$

where in and out denote inlet and outlet of a given artery, respectively.

1.5 Inflow boundary condition

Since venous return to the heart is not modeled, the inflow boundary conditions represent the outflow from the left heart ventricle. This is modeled based on the works of Suga et al. (9,10), and Danielsen and Ottesen (11). The pressure in the left ventricle, P_{lv}, is described using a time-varying elastance function $E_{lv}(t)$:

$$P_{lv} = E_{lv}(t)(V_{lv}(t) - V_0), \qquad \textbf{(9)}$$

where $V_{lv}(t)$ is the ventricular volume at time t and V_0 is the volume of the left ventricle at zero transmural pressure. According to (11), the function $E_{lv}(t)$ may be expressed as follows:

$$E_{lv}(t) = E_{\min}\big(1 - \phi(t)\big) + E_{\max}\phi(t), \qquad \textbf{(10)}$$

where the parameters $E_{\min}$ and $E_{\max}$ are minimal and maximal values of the elastance function $E_{lv}(t)$. Function ϕ is defined by the following equation:

$$\phi(t) = \begin{cases} \alpha \sin\left(\frac{\pi t}{t_m}\right) + \beta \sin\left(\frac{2\pi t}{t_m}\right) \text{ for } 0 \le t < t_m \\ 0 \text{ for } t_m \le t < T \end{cases} \qquad \textbf{(11)}$$

where T is the heart period, t_m denotes the time to the onset of constant (minimal) elastance, and parameters α and β are responsible for the shape of the $\phi(t)$. Additionally, α and β must be chosen so that $\max\limits_{t\in[0,T]} \phi\,(t) = 1$.

The work of the left ventricle can be divided into four stages. We will begin with isovolumic relaxation. During this phase, the pressure in the left ventricle decreases. When P_{lv} is smaller than the pressure in the left atrium, P_{la}, then the mitral valve opens.

In the next phase (ventricular filling) blood flows from the left atrium to the left ventricle. This flow, Q_{la}, is described by the following equation:

$$\frac{dQ_{la}}{dt} = \frac{1}{L_{la}}(P_{la} - P_{lv}) - \frac{R_{la}}{L_{la}} Q_{la}. \qquad \textbf{(12)}$$

Parameter L_{la} is an inertia term, and R_{la} describes the resistance against the flow from the left atrium to the left ventricle, caused mainly by the viscous properties of the blood. Simultaneously, due to the inflow of blood into the left ventricle, V_{lv} increases, as given by the following equation:

$$\frac{dV_{lv}}{dt} = Q_{la}. \qquad \textbf{(13)}$$

When V_{lv} is greater than the end-diastolic volume V_{ed}, the mitral valve closes, and isovolumic contraction begins.

During this phase, there is no flow between the left atrium and ventricle ($Q_{la} = 0$), and P_{lv} increases. When P_{lv} is greater than the pressure in the ascending aorta, P_a, the aortic valve opens, and the last phase of the cycle (ventricular ejection) begins.

The flow between the left ventricle and aorta is expressed by an equation similar to equation (11), namely:

$$\frac{dQ_{lv}}{dt} = \frac{1}{L_{lv}}(P_{la} - P_a) - \frac{R_{lv}}{L_{lv}}Q_{lv}. \tag{14}$$

The pressure P_a is taken directly from the 1D model of the arterial tree. The volume V_{lv} decreases according to the following equation:

$$\frac{dV_{lv}}{dt} = -Q_{lv}. \tag{15}$$

At the end of this phase, some amount of blood, V_b, returns from the ascending aorta to the left ventricle, which is associated with the negative value of Q_{lv} (backflow). V_b is given by the following equation:

$$V_b = \int_{t^*}^{t} |Q_{lv}|, \quad \text{for} \quad t > t^*, \tag{16}$$

where t^* denotes the moment, when Q_{lv} becomes negative. At the time t, when $V_b > \overline{V_b}$ we end the last phase by setting $Q_{lv} = 0$, and then the cycle repeats.

1.6 Outflow boundary conditions

To model blood flow in the small arteries and arterioles downstream from the terminal arteries in our vascular model, we use the three-element Windkessel model (12,13):

$$R_1 R_2 C_T \frac{dQ_{\text{end}}(t)}{dt} = R_2 C_T \frac{dP_{\text{end}}(t)}{dt} + (P_{\text{end}}(t) - P_{\text{term}}) - (R_1 + R_2)Q_{\text{end}}(t) \tag{17}$$

where R_1, R_2 are proximal and distal resistances, respectively, C_T is the compliance of the given terminal vascular branch, and P_{term} is the reference terminal pressure. We assume that $R_1/R_T = 0.2$, where $R_T = R_1 + R_2$ is the total terminal resistance (5,14). The assumed values of R_T and C_T are shown in Table 1. We personalize the model by applying the scaling factors: S_C for compliances and S_R for resistances, see (15) for more details.

2 Default Parameter Values

For our baseline model, we employed parameter values representative of a 25-year-old male with a height of 175 cm. The values of these parameters are provided in Table 2.

Table 2 Default parameter values considered in the cardiovascular model.

Parameter	Unit	Nominal value	Reference	Studied in the sensitivity analysis	Sampling range for sensitivity analyses (± 10%)
μ	$\frac{\text{g}}{\text{cm}^3}$	1.04	(13)	No	-

ρ	$\frac{cm^2}{s}$	0.04	(13)	No	-
P_0	mmHg	97	(14)	No	-
k_1	$\frac{g}{s^2 \cdot cm}$	$3 \cdot 10^6$	(16)	Yes	$[2.7 \cdot 10^6, 3.3 \cdot 10^6]$
k_2	$\frac{1}{cm}$	-13.5	(7)	Yes	[-12.15, -14.85]
k_3	$\frac{g}{s^2 \cdot cm}$	$5.36 \cdot 10^5$	Computed for age=25 from (7)	Yes	$[4.82, 5.90] \cdot 10^5$
a	$-$	0.9	(11)	No	-
b	$-$	0.25	(11)	No	-
$E_{\max}$	$\frac{mmHg}{ml}$	2.5	(11)	Yes	[2.25, 2.75]
$E_{\min}$	$\frac{mmHg}{ml}$	0.049	(11)	Yes	[0.0441, 0.0539]
t_m	s	0.45	(11)	Yes	[0.405, 0.495]
T	$\frac{1}{s}$	0.8	assumed	Yes	[0.72, 0.88]
V_{ed}	ml	127	(11)	Yes	[114, 140]
V_b	ml	2	(11)	Yes	[1.8, 2.2]
V_0	ml	10	(11)	Yes	[9, 11]
$\overline{V_b}$	ml	2	(11)	No	-
R_{lv}	$\frac{mmHg \cdot s}{ml}$	0.0334	(11)	Yes	[0.03, 0.037]
L_{lv}	$\frac{mmHg \cdot s^2}{ml}$	0.000416	(11)	Yes	[0.0003744, 0.0004576]
R_{la}	$\frac{mmHg \cdot s}{ml}$	0.000089	(11)	Yes	$[8.0 \cdot 10^{-5}, 9.8 \cdot 10^{-5}]$
L_{la}	$\frac{mmHg \cdot s^2}{ml}$	0.00005	(11)	Yes	$[4.5 \cdot 10^{-5}, 5.5 \cdot 10^{-5}]$
P_{la}	mmHg	5	(11)	Yes	[4.5, 5.5]

S_R	−	1	assumed	Yes	[0.9, 1.1]
S_C	−	1	assumed	Yes	[0.9, 1.1]
P_{term}	mmHg	15	(2)	No	-

References

1. Poleszczuk J, Debowska M, Dabrowski W, Wojcik-Zaluska A, Zaluska W, Waniewski J. Patient-specific pulse wave propagation model identifies cardiovascular risk characteristics in hemodialysis patients. PLoS Comput Biol. 2018;14(9):1–15.

2. Poleszczuk J, Debowska M, Dabrowski W, Wojcik-Zaluska A, Zaluska W, Waniewski J. Subject-specific pulse wave propagation modeling: Towards enhancement of cardiovascular assessment methods. PLoS ONE. 2018;13(1):1–17.

3. Olufsen MS, Peskin CS, Kim WY, Pedersen EM, Nadim A, Larsen J. Numerical simulation and experimental validation of blood flow in arteries with structured-tree outflow conditions. Ann Biomed Eng. 2000;28(11):1281–99.

4. Stergiopulos N, Young DF, Rogge TR. Computer simulation of arterial flow with applications to arterial and aortic stenoses. J Biomech. 1992 Dec 1;25(12):1477–88.

5. Alastruey J, Parker KH, Peiró J, Byrd SM, Sherwin SJ. Modelling the circle of Willis to assess the effects of anatomical variations and occlusions on cerebral flows. J Biomech. 2007 Jan 1;40(8):1794–805.

6. Ottesen JT, Olufsen MS, Larsen JK, Olufsen MS. 5. Modeling Flow and Pressure in the Systemic Arteries. In: Applied Mathematical Models in Human Physiology. Society for Industrial and Applied Mathematics; 2004. p. 91–136. (Mathematical Modeling and Computation).

7. Charlton PH, Mariscal Harana J, Vennin S, Li Y, Chowienczyk P, Alastruey J. Modeling arterial pulse waves in healthy aging: a database for in silico evaluation of hemodynamics and pulse wave indexes. Am J Physiol Heart Circ Physiol. 2019 Nov 1;317(5):H1062–85.

8. Drzewiecki G, Hood R, Apple H. Theory of the oscillometric maximum and the systolic and diastolic detection ratios. Ann Biomed Eng. 1994;22(1):88–96.

9. Suga H, Sagawa K, Kostiuk DP. Controls of ventricular contractility assessed by pressure-volume ratio, Emax1. Cardiovasc Res. 1976 Sep 1;10(5):582–92.

10. Suga H, Sagawa K, Shoukas AA. Load independence of the instantaneous pressure-volume ratio of the canine left ventricle and effects of epinephrine and heart rate on the ratio. Circ Res. 1973 Mar;32(3):314–22.

11. Ottesen JT, Olufsen MS, Larsen JK, Danielsen M, Ottesen JT. 6. A Cardiovascular Model. In: Applied Mathematical Models in Human Physiology. Society for Industrial and Applied Mathematics; 2004. p. 137–55. (Mathematical Modeling and Computation).

12. Westerhof N, Elzinga G, Sipkema P. An artificial arterial system for pumping hearts. J Appl Physiol. 1971 Nov;31(5):776–81.

13. Blanco PJ, Watanabe SM, Dari EA, Passos MARF, Feijóo RA. Blood flow distribution in an anatomically detailed arterial network model: criteria and algorithms. Biomech Model Mechanobiol. 2014 Nov 1;13(6):1303–30.

14. Stergiopulos N, Young DF, Rogge TR. Computer simulation of arterial flow with applications to arterial and aortic stenoses. J Biomech. 1992 Dec 1;25(12):1477–88.

15. Wołos K, Pstras L, Debowska M, Dabrowski W, Siwicka-Gieroba D, Poleszczuk J. Non-invasive assessment of stroke volume and cardiovascular parameters based on peripheral pressure waveform. PLOS Comput Biol. 2024 Apr 18;20(4):e1012013.

16. Mynard JP, Smolich JJ. One-Dimensional Haemodynamic Modeling and Wave Dynamics in the Entire Adult Circulation. Ann Biomed Eng. 2015 Jun 11;43(6):1443–60.
